# High-performance printable 2.4 GHz graphene-based antenna using water-transferring technology

**DOI:** 10.1080/14686996.2019.1653741

**Published:** 2019-08-20

**Authors:** Weijia Wang, Chao Ma, Xingtang Zhang, Jiajia Shen, Nobutaka Hanagata, Jiangtao Huangfu, Mingsheng Xu

**Affiliations:** a College of Information Science and Electronic Engineering, State Key Laboratory of Silicon Materials, Zhejiang University, Hangzhou, China; b College of Information Science and Electronic Engineering, Zhejiang University, Hangzhou, China; c Key Laboratory of Yarn Materials Forming and Composite Processing Technology of Zhejiang Province, Jiaxing University, Jiaxing, China; d Nanotechnology Innovation Station, National Institute for Materials Science, Tsukuba, Japan

**Keywords:** Graphene, printing, water-transferring, antenna, three-dimensional substrates, 40 Optical, magnetic and electronic device materials, 105 Low-Dimension (1D/2D) materials, 204 Optics / Optical applications, 201 Electronics / Semiconductor / TCOs

## Abstract

Liquid-phase exfoliated graphene sheets are promising candidates for printing electronics. Here, a high-performance printed 2.4 GHz graphene-based antenna is reported. Graphene conductive ink prepared by using liquid-phase exfoliation process is printed onto a water-transferable paper by using blade printing technique, which is then patterned as dipole antenna and transferred onto a target substrate. The fabricated dipole antenna (43 × 3 mm), exhibiting typical radiation patterns of an ideal dipole antenna, achieves −10 dB bandwidth of 8.9% and a maximum gain of 0.7 dBi. The printed graphene-antennas satisfy the application requirements of the Internet of Things and suggest its feasibility of replacing conventional metallic antennas in those applications.

## Graphical abstract

**Figure:**
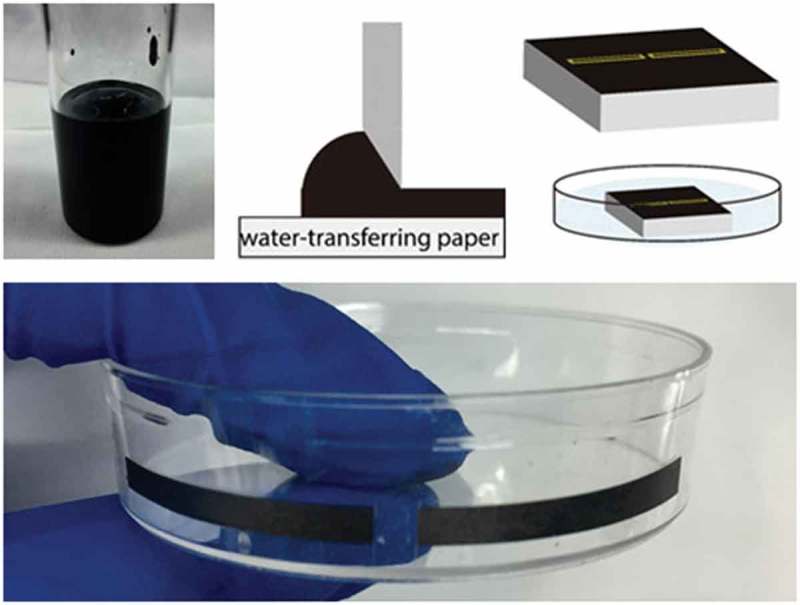

## 1. Introduction

Printed electronics have attracted great interest in recent years [1–4]. It has a wide range of applications, such as antennas [5–8], transparent electrodes [9–11], solar cells [12–14], thin-film transistors [15–17], and light-emitting devices [18–20]. The most widely used conductive inks for printed electronics are metal-based inks due to their high electrical conductivity and excellent mechanical properties. However, there are many obvious disadvantages of using metal-based inks. For example, although silver has high conductivity and its oxide is also conductive [21], the price of silver is so high that makes it an unsuitable choice for mass production. Aluminum and copper are much cheaper than silver, but they easily oxidize in ambient environment and form nonconductive oxides [22,23]. One kind of alternatives to metal-based inks is carbon-based materials, of which graphene is the most attractive and prospective one.

Graphene is a type of two-dimensional (2D) materials, in which carbon atoms are arranged in a honeycomb to form a single carbon layer. The high charge mobility of graphene yields its high conductivity. Moreover, its conductivity is frequency independent up to the microwave region [24,25], which is an advantage for radio frequency (RF) applications. Besides, perfect graphene is so dense that even the smallest atoms such as helium cannot permeate it, and it is chemically stable with high mechanical strength and flexibility [26,27]. On the other hand, the development of graphene exfoliation techniques from graphite in recent years makes it possible to produce graphene-based inks in large scale and at low cost [28,29], which is necessary for mass production of printed electronics.

There are a few recent reports on printed graphene-based antennas, showing potentials for practical applications [7,8,30–36]. However, the reported graphene-based antennas mostly worked at megahertz frequencies (MHz), which indicates that they had a size scale of decimeters [7,8,30–32]. This is too large for portable devices. Furthermore, most of the previously reported graphene-based antennas had a relatively low gain [7,30,31], and the reported graphene-based antennas were all printed directly onto substrates. Hence, a flat substrate is required in such a process, and the direct printing limits its applications in the field of Internet of Things (IoT) where conformal antennas are much preferred to uneven and three-dimensional (3D) objects.

In this work, we report a new process to fabricate printed graphene-based antenna with a high gain and small dimensions. The graphene-based antenna is printed on a water-transferable paper by blade printing and then transferred onto the surface of a target substrate. The fabricated dipole antenna with small dimensions achieves −10 dB bandwidth of 2.297–2.510 GHz (8.9%) with a maximum gain of 0.7 dBi. Such performance of our antenna satisfies its potential applications in the field of IoT.

## 2. Experimental details

**Figure 1.**
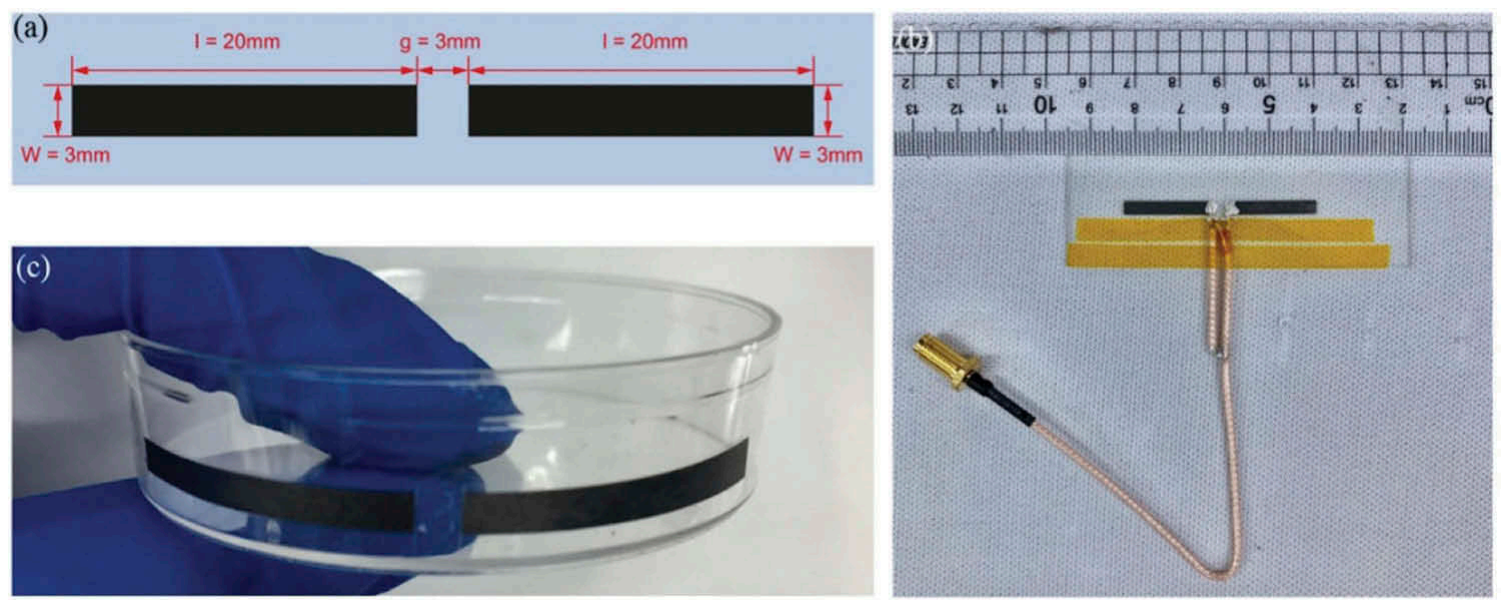
(a) Geometry of designed graphene dipole antenna. Photos of (b) printed graphene-based dipole antenna on the surface of a glass slide with a folded balun, where silver conductive paste was used to feed power into the graphene-based antenna and two yellow stick tapes were used to fix the folded balun onto the substrate, and (c) graphene-based dipole antenna printed on curved surface of a plastic Petri dish using our water-transfer.

The graphene-based dipole antenna was designed and simulated using HFSS (High-Frequency Structure Simulator). Each pole of the antenna has the length of 20 mm and a width of 3 mm. The gap between the two poles is 3 mm (Figure 1).

**Figure 2.**
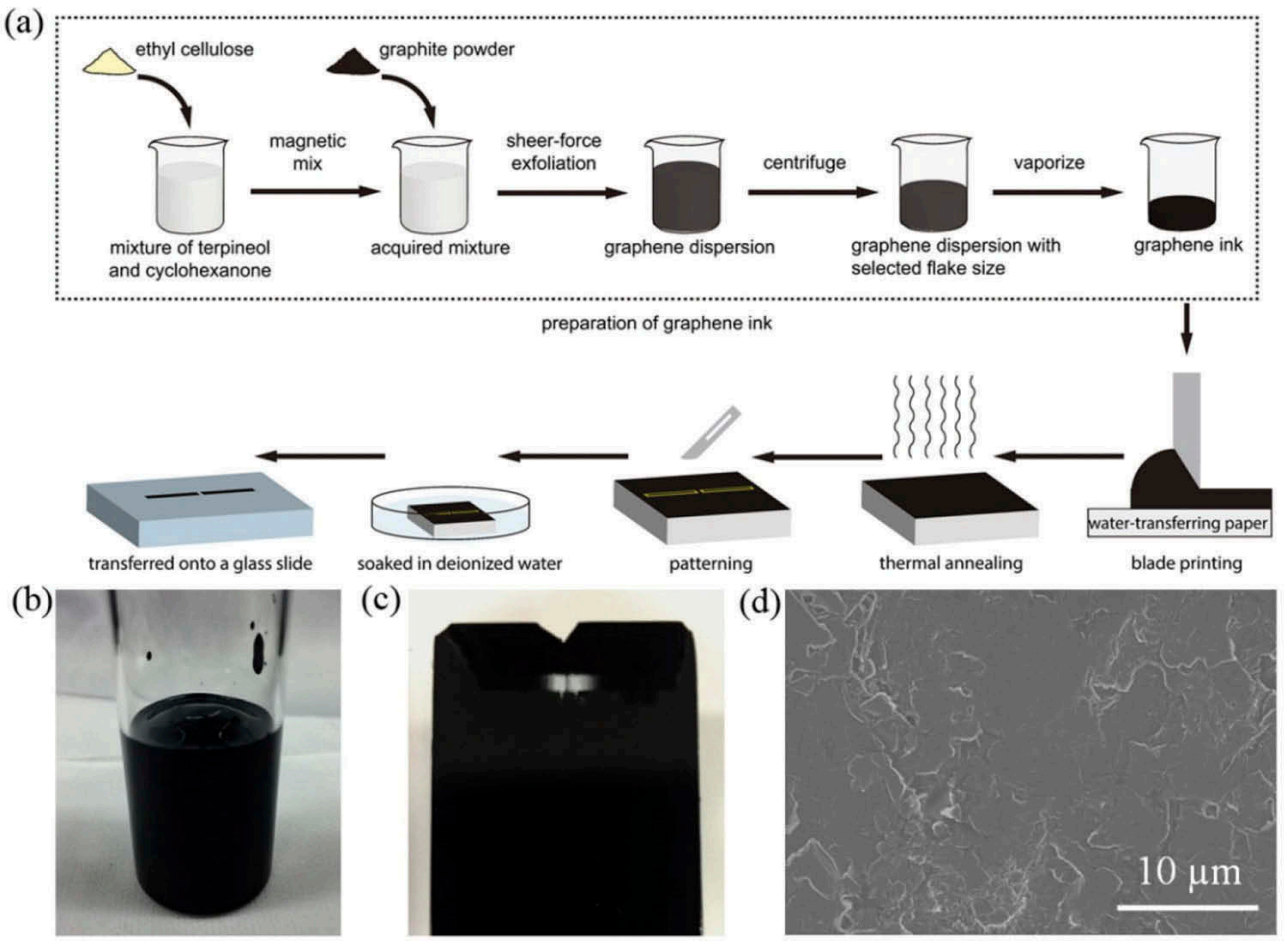
(a) Schematic of the preparation of graphene-based ink, blade printing and transfer of graphene antenna. (b) Photo of prepared graphene ink. (c) Photo of printed graphene layer on water-transferable paper. (d) Scanning electron microscopy image of printed graphene layer.

To prepare the graphene ink for blade printing (Figure 2), ethyl cellulose was added into the mixture of cyclohexanone and terpineol, and dispersed by using a magnetic mixer. Then, graphite powder was added into the mixture and sheered to be exfoliated into graphene sheets with different number of graphene layers by using a high shear mixer Silverson L5M (Silverson, UK). In the present work, the graphite was first exfoliated at 8000 rpm for 8 h, and the resultant dispersion was then centrifugated at 5000 rpm for 30 min. After the centrifugation, most of the top dispersion was removed by pipetting off. The acquired graphene dispersion was further heated at a temperature of 80°C to vaporize the solvent and produce sticky graphene ink which is suitable for blade printing. We hereafter call the sheets as graphene. We performed blade printing by using a tape casting coater equipped with an infrared drier. Graphene ink was spread onto a water-transferable paper (Ruixin, China) by blading and such a process resulted in a thick and uniform graphene layer. The thickness of the printed graphene layer can be adjusted by the separation between the blade and the transferable-paper. After the blading, thermal annealing was performed *in* *situ* immediately with the infrared drier to volatilize the solvent. The heating temperature and time were set to 100°C and 30 min. The water-transferable paper with printed graphene layer was sculpted into the designed antenna pattern. Then, it was soaked into deionized water for 5 min to let the printed graphene layer separate from the paper substrate and transferred onto the surface of a target substrate. Thickness of the printed graphene layer was measured by using a profiler Bruker DektakXT (Bruker, USA) and sheet resistance was measured by using a Hall effect system (Lakeshore 7604, USA).

To perform antenna performance measurements, a coaxial wire with Subminiature Version A connector was attached to the poles using silver conductive paste to feed power into the graphene-based antenna. We integrated a folded balun with the coaxial wire to offset the current resulting from the outer conductor of the coaxial wire. A vector network analyzer Rohde & Schwarz ZNB20 (Rohde & Schwarz, Germany) was employed to measure reflection coefficient (S_11_) of the graphene-based antenna. The radiation patterns of the antenna were measured in an anechoic chamber. The graphene antenna on the glass slide was fixed on a rotary table, with a horn antenna HD-26SGAH15N (Hengda, China) on another rotary table as the receiver antenna. Measurements were performed once per degree in rotation.

## 3. Results and discussion

There are many basic antenna designs, of which a dipole antenna is the most classical and widely used one. It has a simple structure and good performance. As many IoT protocols such as 802.15.4 (Zigbee, Thread), Bluetooth, and ANT operate at 2.4 GHz, our dipole antenna is designed to operate at this frequency. Thus, each pole of the antenna has the length of 20 mm and a width of 3 mm, and the gap between the two poles is 3 mm (Figure 1(a)). The simulated input impedance and radiation efficiency of the antenna are 55 – j2.3 Ω and 95.5%, respectively.

By exploiting blade printing, we can easily produce thick and quite uniform films, which is depicted in Figure 2(c,d). The thermal annealing temperature of printed graphene film was at 100°C in the present study, which is strikingly different from the previously reported 350°C [8]. Our low-temperature annealing is compatible with heat-sensitive substrates such as flexible paper and polyethylene terephthalate. Different from previous reports on directly printing graphene ink on a flat substrate, our process composed of the printing of graphene ink onto a water-transferrable paper and a transferring of the printed graphene layer is suitable for the fabrication of graphene antenna onto an arbitrary substrate, not only flat substrates, but also 3D and uneven objects (Figure 1(c)). This significantly expands the applications of printed graphene antenna. In the present study, we tailored the sheet resistance of our printed graphene layer and it is about 2.6 Ω/□ for the graphene layer with a thickness of 35 um for the dipole antenna. Please note that the sheet resistance of the graphene layer is highly dependent on the preparation of graphene ink, the formation of graphene layer, and thickness of the formed graphene layers. By tuning the above factors, the sheet resistance of our patterned graphene layer is in the range of 0.8–400 Ω/□.

**Figure 3.**
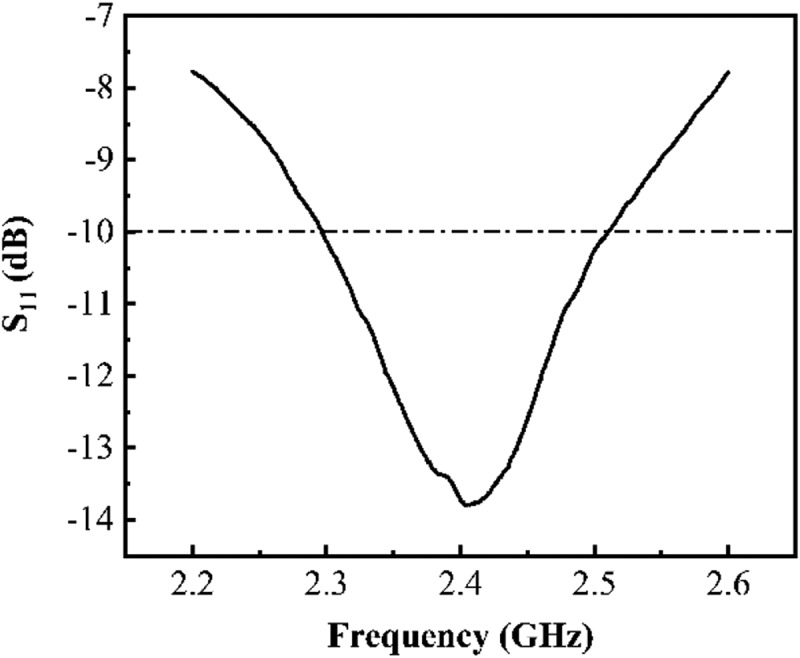
Measured reflection coefficient (S_11_) of printed graphene-based dipole antenna on a glass slide.

We measure the reflection coefficient (S_11_) of our graphene-based antenna and it is found that the measured S_11_ has the minimum value of −13.79 dB at 2.407 GHz (Figure 3), which suggests that our graphene-based antenna operates at the desired frequency and is matched well with the folded balun. Furthermore, the −10 dB bandwidth of the antenna ranges from 2.297 GHz to 2.51 GHz (8.9%). The result suggests that our graphene-based antenna has a fairly wide bandwidth.

**Figure 4.**
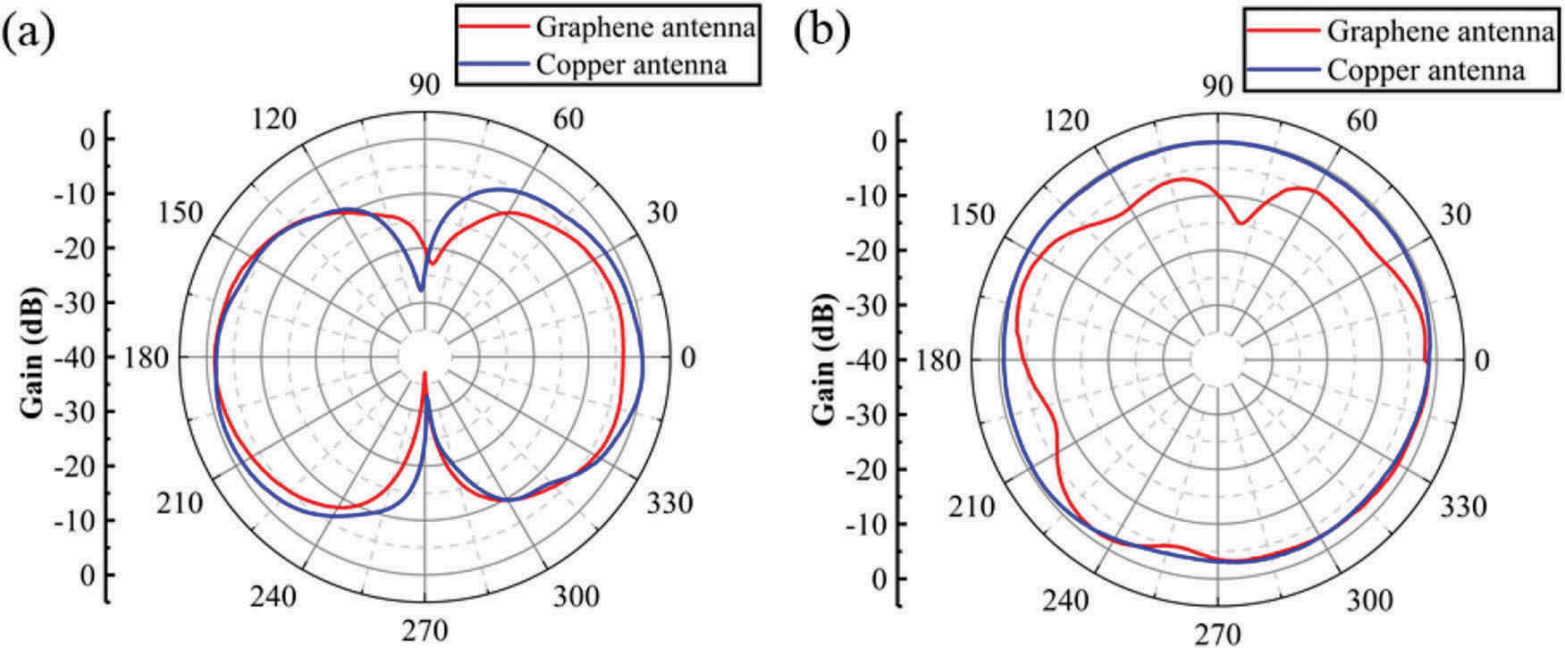
(a) Normalized E-plane radiation patterns of the printed graphene-based dipole antenna and the copper antenna of the same design on a glass slide. (b) Normalized H-plane radiation patterns of the printed graphene-based dipole antenna and the copper antenna of the same design on a glass slide.

We measure the radiation patterns of the graphene-based antenna and compare it with a copper antenna of the same design as the graphene-based antenna. Figure 4(a,b) show the normalized E-plane and H-plane radiation patterns of the printed graphene antenna and the copper antenna at 2.4 GHz, respectively. The radiation patterns of our graphene-based antenna agree with that of the copper antenna in both the E-plane and H-plane, exhibiting the typical dipole antenna radiation patterns. The maximum gain of the graphene-based antenna at 2.4 GHz is measured to be 0.7 dBi, better than those of most previously printed graphene antennas with dipole structures (Table 1). The high gain of an antenna enables a longer reading range as discussed below.

**Table 1.** Performance comparison of typical printed graphene antennas with dipole structures.

Ref.	Operating frequency	Antenna dimensions	Gain
[7]	870 MHz	92×25 mm	−4 dBi
[30]	960 MHz	141×3.5 mm	−0.6 dBi
[31]	889 MHz	143×3 mm	−2.18 dBi
This work	2.4 GHz	43×3 mm	0.7 dBi

On the basis of the parameters of the antenna, we can calculate the theoretical maximum reading range of the antennas when attached to single-chip transceivers according to the modified Friis transmission equation [37]﻿.

$P_{r}=P_{t}\cdot \frac{G_{t}\cdot G_{r}\cdot \lambda^{2}}{{\left(4\pi R\right)}^{2}}\cdot \left(1-{\left|\Gamma_{t}\right|}^{2}\right)\cdot \left(1-{\left|\Gamma_{r}\right|}^{2}\right)$

where $P_{r}$ is received power, $P_{t}$ is transmitted power, $G_{t}$ is the gain of transmitter antenna, $G_{r}$ is the gain of receiver antenna, λ is the wavelength, R is the distance between the transmitter antenna and receiver antenna, $\Gamma_{t}$ is the reflection coefficient of transmitter antenna, and $\Gamma_{r}$ is the reflection coefficient of receiver antenna. We assume a situation where the two nodes, separately equipped with our graphene-based antenna and a single-chip 2.4 GHz highly sensitive transceiver such as nRF52840 (Nordic, Norway) [38] which has protocol support for Bluetooth 5, Bluetooth mesh, Thread, Zigbee, 802.15.4, ANT, and 2.4 GHz proprietary stacks communicate with each other. The maximum output power and the minimum sensitivity of nRF52840 are 8 and −103 dBm, respectively. Consequently, the theoretical maximum reading range can reach as far as 3973 m which matches well with the long reading range demonstrated by the nRF52840 chip. Please note that the reading range of an antenna is also dependent on the transmission power and sensitivity of individual chips. Such a long reading range is sufficient for a wide range of IoT applications, including wearable electronics, smart home, remote controlling, assets tracking, package tracking, and industrial mesh networks. The low fabrication cost, the high gain, the small dimensions yielded by GHz operating frequency, and the capability to be conformal with arbitrary substrates originated from the water-transferring technology are four preferred advantages of the graphene-based antenna for IoT and other relevant applications.

## 4. Conclusions

We have demonstrated the fabrication of high-performance 2.4 GHz graphene-based antenna by printing and using water-transferrable paper. Such a low-temperature and environment-friendly process is suitable for scalable production of graphene-based antennas. Our small-sized graphene dipole antenna has −10 dB bandwidth of 2.297–2.510 GHz (8.9%) with a maximum gain of 0.7 dBi. The performance of our graphene antenna satisfies the application requirements of IoT sensing and suggests its feasibility of replacing conventional metallic antennas in those applications.
